# Comparative transcriptional profiling of the limbal epithelial crypt demonstrates its putative stem cell niche characteristics

**DOI:** 10.1186/1471-2164-11-526

**Published:** 2010-09-29

**Authors:** Bina B Kulkarni, Patrick J Tighe, Imran Mohammed, Aaron M Yeung, Desmond G Powe, Andrew Hopkinson, Vijay A Shanmuganathan, Harminder S Dua

**Affiliations:** 1 Division of Ophthalmology and Visual Sciences, B-Floor, Eye & ENT Building Queen's Medical Centre, Derby Road, Nottingham, UK; 2 Department of Pathology, A-Floor, West Block, Queen's Medical Centre, Derby Road, Nottingham, UK

## Abstract

**Background:**

The Limbal epithelial crypt (LEC) is a solid cord of cells, approximately 120 microns long. It arises from the undersurface of interpalisade rete ridges of the limbal palisades of Vogt and extends deeper into the limbal stroma parallel or perpendicular to the palisade. There are up to 6 or 7 such LEC, variably distributed along the limbus in each human eye.

Morphological and immunohistochemical studies on the limbal epithelial crypt (LEC) have demonstrated the presence of limbal stem cells in this region. The purpose of this microarray study was to characterise the transcriptional profile of the LEC and compare with other ocular surface epithelial regions to support our hypothesis that LEC preferentially harbours stem cells (SC).

**Results:**

LEC was found to be enriched for SC related Gene Ontology (GO) terms including those identified in quiescent adult SC, however similar to cornea, limbus had significant GO terms related to proliferating SC, transient amplifying cells (TAC) and differentiated cells (DC). LEC and limbus were metabolically dormant with low protein synthesis and downregulated cell cycling. Cornea had upregulated genes for cell cycling and self renewal such as *FZD7, BTG1, CCNG*, and *STAT3 *which were identified from other SC populations. Upregulated gene expression for growth factors, cytokines, WNT, Notch, TGF-Beta pathways involved in cell proliferation and differentiation were noted in cornea. LEC had highest number of expressed sequence tags (ESTs), downregulated and unknown genes, compared to other regions. Genes expressed in LEC such as *CDH1, SERPINF1, LEF1, FRZB1*, *KRT19, SOD2, EGR1 *are known to be involved in SC maintenance. Genes of interest, in LEC belonging to the category of cell adhesion molecules, WNT and Notch signalling pathway were validated with real-time PCR and immunofluorescence.

**Conclusions:**

Our transcriptional profiling study identifies the LEC as a preferential site for limbal SC with some characteristics suggesting that it could function as a 'SC niche' supporting quiescent SC. It also strengthens the evidence for the presence of "transient cells" in the corneal epithelium. These cells are immediate progeny of SC with self-renewal capacity and could be responsible for maintaining epithelial turn over in normal healthy conditions of the ocular surface (OS). The limbus has mixed population of differentiated and undifferentiated cells.

## Background

**Figure 1:**
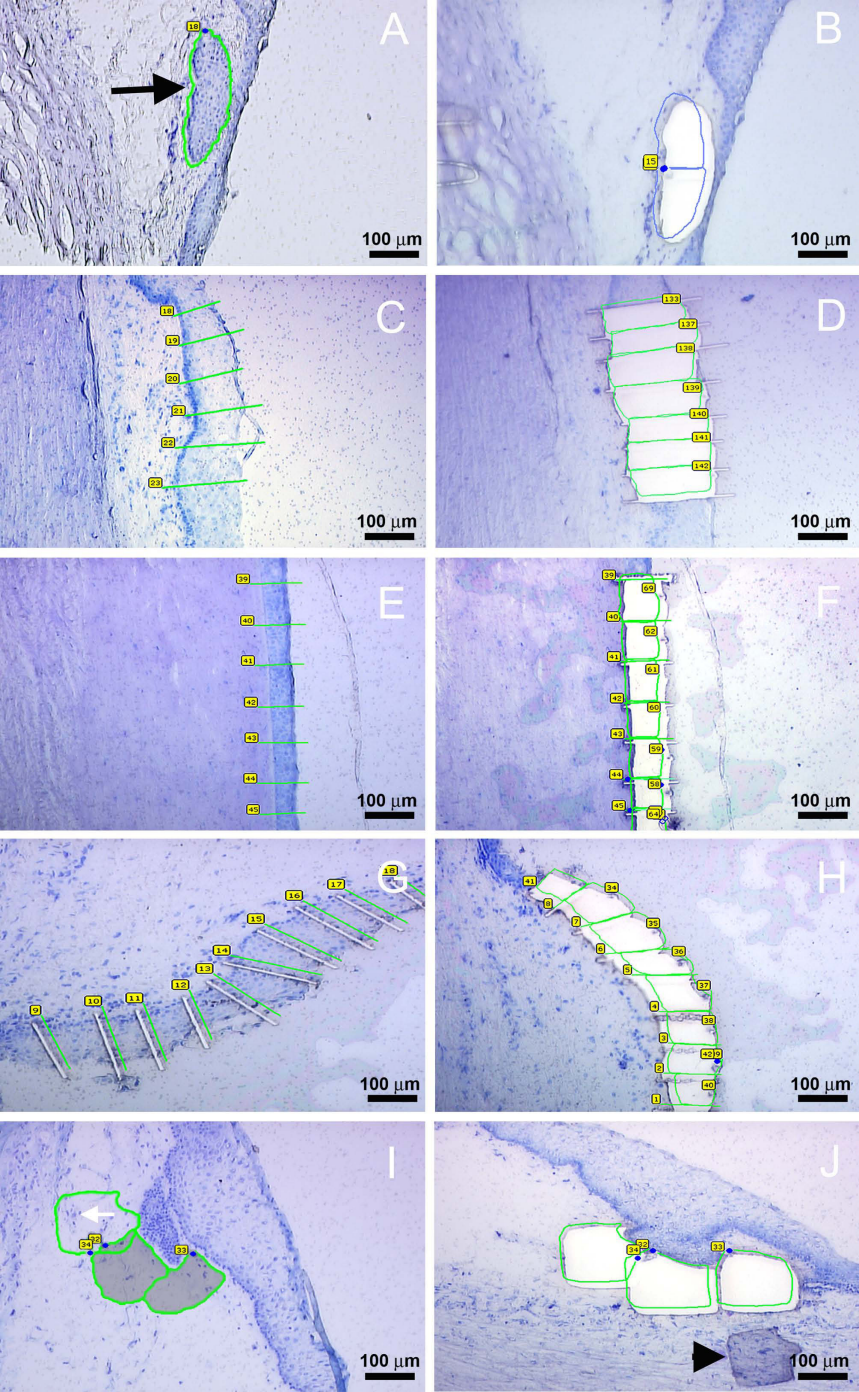
**Laser Microdissection (LMD) of the ocular surface epithelial regions**. The composite shows steps of LMD performed on radial cut section of LEC (A, B,), limbus (C, D), cornea (E, F), conjunctiva (G, H) and LEC stroma (I, J) at 20× magnification (scale bars shown). Figure 1A is of pre LMD LEC, shown with black arrow. Figure 1 I is of LEC stroma with cells shown with white arrow. Prior to LMD, the sections were stained with RNase free Toluidine Blue. Images (A, C, E, I,) are examples of pre LMD OS epithelial region sections with outlines for laser cuts drawn around the tissue. The junctions between the OS epithelial regions were avoided as it has overlapping features of the two adjacent regions. Image (G) shows cut sections of the epithelial regions. Dividing the epithelium into multiple small pieces facilitated effective catapulting of the tissue into the collection cap. Images (B, D, F, H, J) represent examples of post LMD sections of OS epithelium following pressure catapulting of the epithelial pieces. Image J shows a misdirected piece of LEC Stroma which was not captured in the cap but settled down over the adjacent LEC Stroma (black arrow head); such tissue pieces could be recatapulted into the cap with dot LPC laser function.

Corneal transparency is crucial for sight. The corneal epithelium and tear film provide the polished outer surface to the cornea enabling it to function as a refractive surface. It is postulated that the continued supply of the epithelial cells is maintained by the SC at the limbus. Numbers of studies have provided direct and indirect evidence to support this notion. In 1986 Schermer et al proposed the limbal location of corneal stem cells based on keratin expression of corneal epithelial cells [1]. Other studies providing evidence of presence of corneal stem cells at limbus include immunohistochemistry studies with known SC markers [2-4], cell cycling studies characterising the limbal-basal epithelium [5,6] and electron microscopic features of the basal epithelial cells [7]. We have identified a unique structure at the limbus, termed as the limbal epithelial crypt (LEC) [8]. It is a solid cord of cells which extends from the peripheral aspect of the undersurface of interpalisade rete ridges of limbal palisades of Vogt into the limbal stroma (Figure 1A). There are up to 6 or 7 such LEC, variably distributed along the limbus in each human eye. The LEC is analogous to the deep ridges reported in the monkey palm epithelium, where the basal cells of the deep ridges are shown to be the slow cycling stem cells. Similar to the deep location of the ridges in the monkey palm, the deep location of the LEC would offer physical protection to the SC population [9,10]. Our anatomical and immunohistological studies on the LEC have emphasised its potential as a repository of SC and as a putative SC niche (SCN) [11,12], a concept first proposed by Schofield in 1978 [13]. Constituents of the niche include tissue cells, and extra-cellular substrates that sustain the SC and control their self renewal and progenitor potential *in vivo *[14]. The niche provides a specialised microenvironment whereby SC are maintained in a state of quiescence. Cellular quiescence indicates slow cell cycling or growth arrested phase of the cells. In adult SC it protects against environmental stresses and aids in their maintenance. This property was identified in various cell populations such as in the bulge region of hair follicles [15], intestinal [16], haematopoietic [17], muscle satellite SC [18] and also in the limbus [19]. In the hair follicle bulge region two SC compartments have been identified [15]; the quiescent and the activated progenitor cells. The latter regenerates the tissue in homeostatic conditions whereas; quiescent cells act as a reservoir and undergo cell cycling following tissue injury. Several studies have identified possible SC markers in the limbal epithelium, using a mechanical dissection technique [20-22]. This method potentially involves the risk of contamination from surrounding tissue. However, laser microdissection (LMD) has allowed the isolation of a pure population of both limbal and corneal epithelium *in situ *[23]. Studies have also been performed on limbal sub-populations using different techniques including cell cultures [24], collagen adhesiveness [25] and flow cytometry [26]. However such methods involving cellular manipulation can influence gene expression. This transcriptional profiling study of laser microdissected OS epithelial regions (LEC, limbus, cornea, conjunctiva and LEC stroma) demonstrates the average characteristic features of each region rather than of the individual cell populations. Broadly, this study highlights the presence of undifferentiated and quiescent SC in the LEC and "transient cells" or activated progenitor cells and differentiated cells in the cornea [27]. The gene expression of limbus is suggestive of presence of quiescent cell population with differentiated suprabasal epithelial cells. Our study provides evidence to support the hypothesis that the LEC is the reservoir of the SC and could serve as a SC niche at the human OS.

## Results and Discussion

Transcriptome analysis of OS regions; 1) LEC, 2) limbus, 3) cornea, 4) conjunctiva and 5) LEC stroma was performed on four biological replicates for each region, processed from four pairs of cadaver human eyes. Poor quality raw data from a corneal and a conjunctival replicate were excluded from the analysis. Following normalisation and filtration, a data set of 4574 genes for 18 samples was created.

### Differentially Expressed Gene Lists

**Figure 2:**
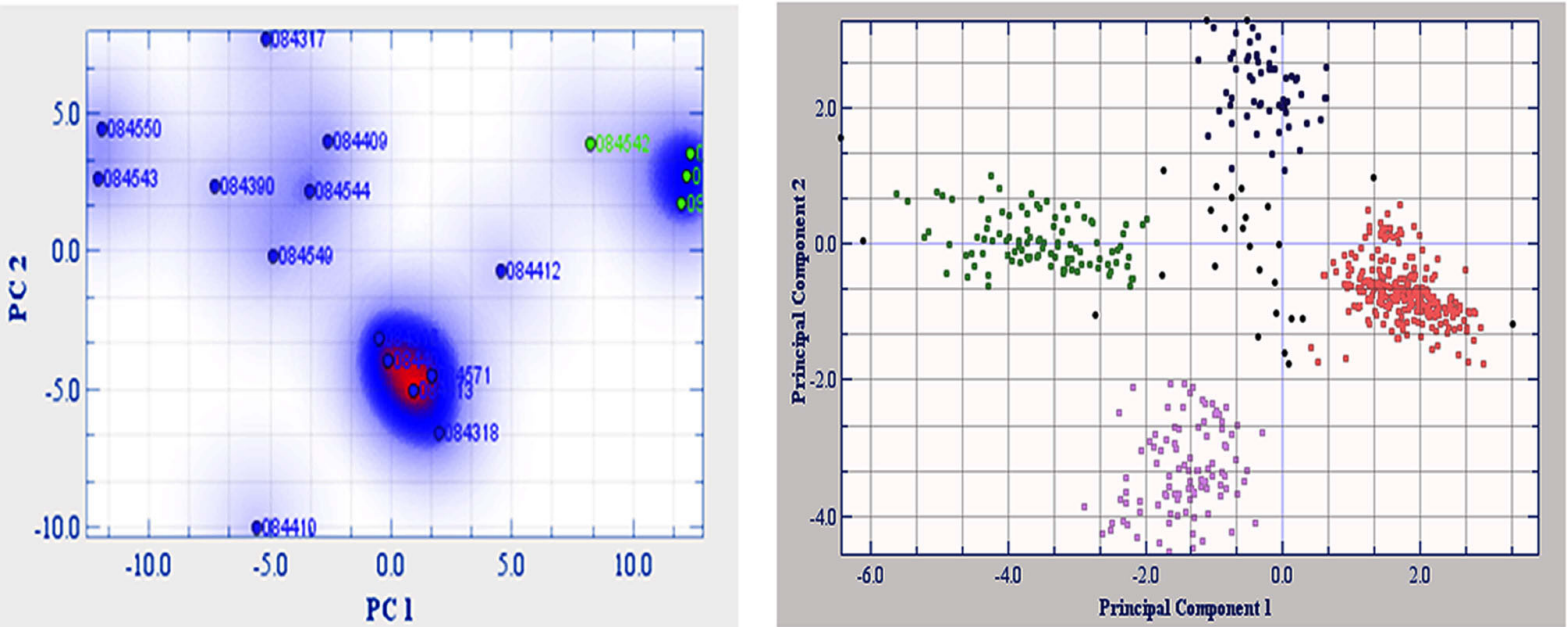
**Principal Component Analysis (PCA) plot of microarray samples and genes**. Left image shows PCA of LEC *vs *ALL samples following feature subset analysis performed on Jexpresspro software. The LEC samples are represented as green dots and rest of the samples as blue dots. LEC biological replicates are seen to cluster separately from rest of the samples indicating differences between LEC and other sample groups but similarity or reproducibility between LEC replicates. Right image shows PCA of differentially expressed genes (527) between LEC and cornea. These are clustered into four distinct coloured groups according to the density. The red group has the highest density and blue the lowest, black dots in the centre are unclustered genes.

Principal Component Analysis (PCA) grouped the biological replicates for each OS region. Figure 2 (left) shows LEC replicates segregated from other OS regions. This demonstrated similarity and reproducibility amongst the LEC biological replicates. Notably, PCA of differentially expressed genes between LEC and cornea generated distinct gene clusters (figure 2, right) as these two regions are at the opposite ends of the epithelial differentiation spectrum. The differentially expressed gene list for each OS region demonstrated highest upregulated gene expression in the cornea and most downregulated genes in LEC. Our findings showed that cornea was the most biologically active zone, whereas the LEC was a metabolically dormant zone (table 1).

**Table 1:** Differentially expressed gene lists of OS epithelial regions

Ocular Regions	Total genes	Up-reg genes	Down-reg genes	DAVID IDs		IPA IDs	
				**mapped**	**unmapped**	**mapped**	**unmapped**

LEC	704	95	609	483	115	548	156

Limbus	463	169	294	294	79	355	106

Cornea	1649	1237	412	939	192	1273	370

LEC Stroma	329	113	216	234	52	266	70

Conj	400	281	119	295	77	305	95

In DAVID 2008 and Ingenuity Pathway Analysis the mapped gene IDs were analysed and the unmapped gene IDs were excluded as these were either unknown genes, ESTs, sequences or duplicated genes.

### Gene Ontology (GO)

**Figure 3:**
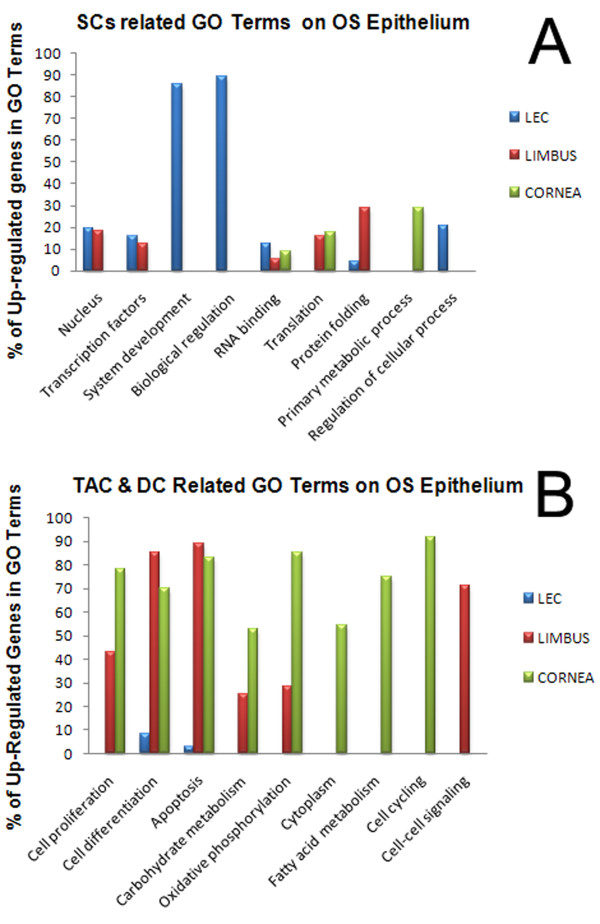
**Composite image of Gene Ontology graphs**. Bar graph (A) shows statistically significant (p value ≤ 0.05) SC related GO terms in LEC, Limbus and cornea. Bar graph (B) represents statistically significant (p value ≤ 0.05) TAC and DC related GO terms in LEC, limbus and cornea. In both graphs A and B the x and y axes indicate the GO terms related to gene functions and percentages of differentially expressed upregulated genes in each category respectively. Absent bar chart for particular GO term in a region was either because of downregulated or absent gene expression.

Considering the model of corneal epithelial regeneration [12,28-30], comparative GO profiling of LEC, limbus and cornea, was performed with the GO terms categorised according to specific functions [31] related to stem, transient amplifying (TAC) and differentiated cells (DC) [32,33]. SC related GO terms enriched in LEC and limbus were *Transcription from RNA polymerase II promoter*, *regulation of transcription from RNA polymerase II promoter *and *RNA binding. *Compare analysis performed on Ingenuity pathway analysis (IPA), characterised the 'tissue specific' nature of each region. LEC was found to be enriched for undifferentiation processes such as S*ystem development *(4.2E-04), including *spermatogenesis *(*SLC12A2*), *development of haematopoietic progenitor cells *(*EGR1*) and *nervous system development *(LEF1, EGR1, PARK2, SLC12A2). This suggests that the LEC is a more undifferentiated and stem-like region than the adjoining limbus. Also down regulation of genes related to TAC terms, such as *cell proliferation *and *apoptosis *in LEC demonstrates its primitive features, unlike limbus. Meta-analysis performed by Edwards et al on SC microarray data sets have identified GO terms related to quiescent and proliferating SC [33]. Based on this study we have found that LEC was specifically enriched for quiescent SC (QSC) related terms such as *Biological Regulation*, (p value: 4.0E-2), and *Regulation of cellular processes *(p value: 5.0E-2) (figure 3A). However limbus [*Protein folding *(p value: 2.6E-2)] and cornea [*Primary metabolic process *(p value: 7.1E-5), *Translation *(5.5E-11)] were enriched for PSC related GO terms. Additionally cornea and limbus were enriched for TAC and DC related GO terms (figure 3B).

### Molecular Features of stem/transient cells in OS regions

Adult SC are quiescent or slow cycling, whilst their immediate progeny the "transient cells" (early TAC) and subsequent TAC undergo active cell cycling, proliferation and differentiation [34]. SC related GO terms further studied on DAVID 2008 using functional annotation clustering were: i) transcription factors; ii) self renewal, iii) cell proliferation; iv) cell differentiation; v) negative regulation of cell proliferation; and vi) cell cycling.

i) *Transcription factors *are involved in development and SC functions such as regulating cell fate determination, cell cycling, cell differentiation and response to environment. This GO term was significant only to LEC (p value: 3.50-05) and limbus (p value: 5.70E-04), (figure 3A, table 2). LEF1 has been shown to be crucial in hair follicle patterning [35] epithelial invagination into mesenchyme [36], and in maintenance of SC quiescence [37,38], it was found to be upregulated in LEC. In contrast, the upregulated transcription factors in limbus were involved in cell proliferation and differentiation (table 2). A study on embryonic limbus has demonstrated expression of *SOD2 *(transcription factor and antioxidant) exclusively in limbus from 14 weeks of development [39]. However our microarray study had noted upregulated expression of SOD2 in LEC, limbus and cornea (table 3)

**Table 2:** Candidate genes expressed in SC related GO terms

LEC		Limbus		Cornea	
**Accession No**.	**Gene Symbol** **(Foldchange)**	**Accession No**.	**Gene Symbol** **(Foldchange)**	**Accession No**.	**Gene Symbol** **(Fold change)**

**Transcription factors (GO ID 0006366)**					

NM_001964	EGR1 (1.8)	NM_000024	ADRB2 (2.7)	-	-

NM_016269	LEF1 (1.3)	NM_006885	ZFHX3 (1.5)	-	-

NM_004492	GTF2A2 (4.2)	NM_001537	HSBP1 (1.7)	-	-

NM_004290	RNF14 (5.1)	NM_006237	POU4F1 (1.9)	-	-

NM_000636	SOD2 (3.5)	NM_002165	ID1 (1.5)	-	-

NM_005997	VPS72 (5.5)	NM_000636	SOD2 (1.6)	-	-

**Self Renewal**					

NM_004360	CDH1 (3.1)	NM_001537	HSBP1 (1.7)	NM_001731	BTG1 (3.0)

NM_016269	LEF1 (1.3)	-	-	NM_004060	CCNG1 (1.7)

-	-	-	-	NM_002592	PCNA (8.8)

-	-	-	-	NM_033632	FBW7 (6.1)

-	-	-	-	NM_003908	EIF2S2 (4.9)

-	-	-	-	NM_003507	FZD7 (2.0)

-	-	-	-	NM_001553	IGFBP7 (9.7)

-	-	-	-	BC004241	LAMA4 (2.8)

-	-	-	-	NM_000269	NME1 (4.5)

-	-	-	-	NM_003150	STAT3 (1.7)

-	-	-	-	NM_005524	HES1 (2.1)

**Cell Proliferation (GO ID: 0008283)**					

-	-	NM_002350	LYN (1.2)	NM_001421	ELF4 (1.9)

-	-	NM_004160	PYY (2.4)	NM_002520	NPM1 (8.5)

-	-	NM_000024	ADRB2 (2.7)	NM_153815	RASGRF1 (6.2)

-	-			NM_002272	KRT5 (2.5)

-	-			NM_005154	USP8 (3.2)

-	-			NM_016272	TOB2 (1.8)

**Cell Cycling (GO ID: 0007049)**					

-	-	-	-	NM_001553	IGFBP7 (7.5)

-	-	-	-	NM_000636	SOD2 (4.9)

-	-	-	-	NM_001924	GADD45A (1.6)

-	-	-	-	AL050044	GADD45B (3.1)

-	-	-	-	AL096865	RUNX2 (2.4)

-	-	-	-	NM_015895	GMNN (2.6)

-	-	-	-	NM_021953	FOXM1 (1.9)

-	-	-	-	NM_002520	NPM1 (8.5)

-	-	-	-	NM_002165	ID (3.6)

-	-	-	-	NM_001553	IGFBP7 (9.7)

-	-	-	-	AB037594	ING1 (2.1)

**Table 3:** Candidate genes expressed in GO terms influencing the niche microenvironment, of stem/progenitor cells

LEC		Limbus		Cornea	
**Accession No**	**Gene Symbol**	**Accession No**	**Gene Symbol**	**Accession No**	**Gene Symbol**

**CAM**					

NM_022843	PCDH20 (1.9)	NM_002318	LOXL2 (2.5)	AL109804	SIGLEC1 (1.7)

AL109804	SIGLEC1 (1.8)	-	-	NM_022121	PERP (4.9)

NM_004360	CDH1 (3.1)	-	-	NM_002589	PCDH7 (3.2)

-	-	-	-	NM_004572	PKP2 (3.8)

**Growth factors**					

-	-	NM_007083	NUDT6 (1.7)	NM_014624	S100A6 (5.7)

-	-	-	-	U62317	ECGF1 (2.7)

-	-	-	-	AC005234	TGFA (2.6)

-	-	-	-	NM_004494	HDGF (3.3)

**Cytokines**					

-	-	NM_002350	LYN (1.2)	NM_199193	BRE (3.3)

-	-	-	-	NM_005625	SDCBP (2.4)

-	-	-	-	NM_021709	SIVA1 (3.8)

-	-	-	-	NM_017801	CMTM6 (3.9)

-	-	-	-	NM_004757	SCYE1 (7.9)

-	-	-	-	NM_016951	CKLF (3.9)

-	-	-	-	U52912	LEPR (3.5)

-	-	-	-	NM_000565	IL6R (8.7)

-	-	-	-	AJ271736	IL9R (4.2)

**Antioxidants**					

NM_006406	PRDX4 (4.1)	NM_012094	PRDX5 (1.6)	NM_000454	SOD1 (3.9)

NM_000636	SOD2 (3.5)	NM_000636	SOD2 (1.6)	NM_000636	SOD2 (4.9)

-	-	-	-	NM_002574	PRDX1 (4.3)

-	-	-	-	NM_006793	PRDX3 (1.9)

-	-	-	-	NM_006406	PRDX4 (7.2)

-	-	-	-	NM_012094	PRDX5 (3.0)

-	-	-	-	NM_004528	MGST3 (3.7)

-	-	-	-	NM_018445	SELS (2.0)

-	-	-	-	NM_018833	TAP2 (2.9)

-	-	-	-	NM_000852	GSTP1 (3.7)

**Neurotropins**					

NM_002615	SERPINF1(2.4)	NM_002615	SERPINF1(3.1)	AY029066	Humanin (4.3)

**Immunomodulatory**					

AL109804	SIGLEC1 (1.8)	AL035662	CD40 (1.5)	AF098366	IGHG1 (5.6)

-	-	-	-	NM_030790	ITFG1 (4.4)

-	-	-	-	AL035662	CD40 (2.3)

**Extracellular matrix**					

-	-	NM_002318	LOXL2 (2.5)	BC004241	LAMA4 (2.8)

*ii) Self renewal *is an important feature of SC maintenance, which isabsent in differentiated cells, weakly expressed in quiescent SC but significantly expressed in proliferating progenitor cells such as mesenchymal stem cells (MSC), [40], intestinal crypts and neural crest cells[41,42]. Based on other SC studies such as adult SC [43], MSC [44], neural SC [45] and neural progenitor cells[46] we had found self renewal genes to be predominantly upregulated, in cornea (table 2). This is indicative of the presence of early TACs, (Transient cells), with self-renewal properties in cornea. These cells could sustain healthy corneal epithelium independent of limbal/LEC support. Dua *et al *[27] have demonstrated the long term conservation of 'central islands' of normal corneal epithelium in patients with total limbal stem cell deficiency as determined clinically by in vivo confocal microscopy. Study on cornea of certain mammals by Majo *et al *have demonstrated holoclone capacity of the central cornea and showed that the central corneal epithelium, when transplanted to the limbus, could regenerate corneal epithelium [47]. However Sun et al have refuted this and argued that true corneal epithelial SC are located primarily at the limbus and not in the central cornea [48]. Data from this study supports the latter notion, and further refines the preferential location of SC in the LEC. An ex-vivo study on organ cultured corneas has demonstrated that the central corneal epithelium depicts a regenerative potential in acute wound healing [49]. Some of these differences are clearly species dependent and any oligopotent potential of human central cornea is not yet proven. Data from the above reports can be reconciled by proposing that the SC are primarily located at the limbus/LEC and that the central cornea contains cells that are capable of maintaining a sustained regenerative capacity in the absence of a functional limbus, as well as in the uninjured physiological state. This supports the hypothesis that the transition from a SC to a TAC is not abrupt but that there exists a population of cells between these two stages that have an intermediate potential, which we have termed "transient cells", and can migrate to populate the central cornea[27]. In this study presence of enriched TAC related GO terms with significant gene expression in cell cycling, self renewal, cell proliferation in cornea (table 2) is suggestive of the presence of such "transient cells" with self renewal capacity in this region.

*iii) Cell proliferation *is a property of activated progenitor cells. Upregulated genes for cell proliferation in cornea mainly belonged to TGFB1 and the Ras Oncogene family (table 2). Integrin Beta 1 Binding Protein 1 (ITGB1BP1) involved in disruption of focal cellular adhesion and mobilisation of stem/transient cells via the c-Myc promoter [50] was found to be downregulated in LEC. This gene was further validated by real time PCR. *LYN *a tyrosine kinase molecule involved in cell proliferation of haematopoietic stem cells [51] was also found to be upregulated in limbus.

*iv) Cell differentiation *is a process whereby undifferentiated regenerative cells acquire specialised structural and functional features of mature cells. It was enriched in all the 3 groups. Of the sixty two, cell differentiation genes identified in LEC only five were upregulated (table 2) these were involved in maintaining epithelial cells in an undifferentiated state. However, in limbus of the 43 genes 23 were upregulated, out of which only 3 genes were involved in maintaining the cells in undifferentiated state (*KRT19, SOD2, KRT14*) (table 2). *KRT19 *has been identified in epidermal stem/progenitor cell population with a role in negative regulation of differentiation [52]. Lyngholm, M *et al *identified *SOD2 *as a marker of limbal SC [53], it was found to be upregulated in LEC, limbus and cornea in this study.

In cornea, of 113 genes expressed, 79 were upregulated and were involved in differentiation, including terminal differentiation (table 2). Retinoic acid pathway involved in inhibition of proliferation of corneal TAC [54] was also expressed in cornea (*RXRG, RARRES3, CYP26A1*) (table 2). Epidermal Differentiation Complex (EDC) is a family of S100 related genes crucial for terminal differentiation of human epidermis [55]. Of the 18 known S100 genes for EDCs four were upregulated in cornea and one in limbus (table 2). Down-regulation of all these protein complexes was noted in LEC. Apolipoproteins expressed in differentiated cells were found to be upregulated in cornea and absent or downregulated in the LEC and limbus.

*v) Negative regulation of cell proliferation*, also known as 'cell quiescence', was enriched only in LEC (p value: 1.4E-2), and LEC stroma (p value: 3.5E-2). *SERPINF1 *a secreted Neurotropin with potent antiangiogenic properties upregulated in early passage cells in G0 phase as compared to actively proliferating or senescent cells [56] was also found to be upregulated in LEC and limbus.

*vi) Cell cycling *(*GOTERM_BP_ALL: GO: 0007049) *refers to replication and segregation of genetic material followed by cell division. Cornea was enriched for this GO term (p value: 2.9E-2). However LEC (p value: 2.4E-01) and limbus (P value: 5.6E-02) were not enriched for this GO term. Of the 60 genes expressed in cornea, 55 were upregulated and mainly represented G1, G2/M phase including *cyclins G1, H, C *and *cyclin D *type binding protein.

vii) *Keratins: KRT19 *a known epidermal SC marker [52] was also found to be upregulated in LEC and limbus; hence supporting the evidence of presence of SC in these regions. However limbus also had upregulated expression of *KRT 14 *which is expressed by mitotically active basal cells of stratified epithelium [57] and *KRT 13 *expressed by suprabasal cells of non-cornified stratified epithelia [58]

### Molecular features of OS SC niche components

Adult SC are influenced by their microenvironment or the niche, which regulates their function. Niche components identified in OS regions included cell adhesion molecules (CAMs), growth factors, cytokines, extracellular matrix and secreted proteins like neurotropins (table 3). E-cadherin, a CAM upregulated in LEC was previously shown to anchor the SC to the basement membrane and thus aid in SC maintenance [59] and prevent aging of SC [60]. *SIGLEC1 *a CAM and a cell surface receptor, also upregulated in LEC, has been shown to be involved in epithelial regeneration [61]. Growth factors and cytokines promote proliferation and these were found to be upregulated in cornea (table 3). However *IGFBP2 *(Insulin Growth Factor Binding Protein 2) which is known to influence epidermal regeneration and SC maintenance [62] was found to be upregulated in LEC. Protection of stem and progenitor cells from oxidative stress is crucial for their sustained maintenance. The GO term detoxifier system (*GOTERM_MF_ALL: GO: 0016209*) which confers antioxidant protection, was enriched in LEC (P value: 5.0E-2), cornea (p value: 5.1E-3) and limbus (p value: 4.7E-2). *SOD2 *an antioxidant was found to be upregulated in LEC, limbus and cornea (table 3). It was previously shown to prevent the aging of the SC and their niches in human epidermal keratinocytes [60]. We further validated the expression of SOD2 in these regions with real time PCR.

Unlike the cornea, the stroma of the limbus has been shown to maintain the basal epithelial layer in an undifferentiated condition, preserving the stemness of the SC [63]. Previous studies have shown the presence of a heterogeneous population of cells which include bone marrow derived mesenchymal cells in the limbal stromal region [64] along with the limbal epithelial progenitor cells that have migrated in the process of epithelial mesenchymal transition (EMT) [65]. Similarly we had noted round undifferentiated epithelial and spindle like mesenchymal cells in LEC stroma on histological sections of LEC (marked with white arrow head) (Figure 1I). Therefore we further studied the gene expression profile encoding the GO term "secreted" (extra cellular region GO: 0005576) for its influence on the LEC cells. The absence of CDH1 in LEC stroma, along with activation of LEF1/WNT β-Catenin signalling pathways in LEC could account for EMT which was similarly reported in a previous study on limbal stroma [66]. LEC stroma was enriched for the tissue metalloproteinase inhibitors (*TIMP1, TIMP2*), developmental protein *FLII *(7.2), antioxidants *XPA *(4.57), *DUOX1 *(5.11), member of GDNF family *GFRα4*, and *LMNA *a nuclear envelope matrix protein, all of which have been reported to be involved with SC maintenance in other tissues[67,68].

Unlike epithelium from different regions, we did not compare the LEC stroma with stroma from other regions. This is a limitation of the current work; however, we were able to compare the gene expression of LEC stroma with published data on limbal stroma [63]. The above mentioned data on LEC stroma is supportive of its role in maintenance of the LEC cells in undifferentiated state.

### Canonical pathways on ocular surface

IPA was used to characterise the enriched canonical metabolic and signalling pathways on the OS epithelial regions (table 4).

**Table 4:** Enriched canonical signaling and metabolic pathways in OS epithelial regions

Canonical metabolic pathways	P-Value	No of genes
**LEC**		

Oxidative Phosphorylation	4.52E-17	29

Ubiquinone biosynthesis	8.04E-12	14

Purine metabolism	0.00166	18

Pyrimidine metabolism	0.0274	9

**Limbus**		

Pentose Phosphate Pathway	0.0014	5

Oxidative Phosphorylation	0.00795	7

Valine, Leucine and Isoleucine Biosynthesis	0.00851	2

Alanine and aspartate metabolism	0.0151	3

Glycolysis/gluconeogenesis	0.047	4

**Cornea**		

Oxidative Phosphorylation	3.48E-21	41

Ubiquinone Biosynthesis	2.14E-08	16

Purine metabolism	0.000118	30

Alanine and Aspartate metabolism	0.000869	9

Glutathione metabolism	0.00607	8

Glycolysis/gluconeogenesis	0.00855	12

Lysine biosynthesis	0.0402	2

Citrate cycle	0.042	4

Pyruvate metabolism	0.0451	8

**Canonical Signaling Pathways**		

**LEC**		

NRF2-mediated Oxidative Stress Response	2.29E-05	16

Nucleotide Excision Repair Pathway	3.17E-05	7

Protein Ubiquitination Pathway	2.06E-05	16

Estrogen Receptor Signaling	8.79E-03	8

Glucocorticoid Receptor Signaling	1.81E-02	13

**Limbus**		

NRF2-mediated Oxidative Stress Response	2.81E-02	7

Regulation of actin based motility by Rho	4.51E-02	4

**Cornea**		

NRF2 mediated Oxidative Stress Response	2.51E-04	21

Protein Ubiquitination Pathway	3.09E-07	26

Estrogen Receptor Signaling	3.67E-04	15

Antigen Presentation Pathway	1.29E-03	7

VDR/RXR Activation	6.31E-03	10

Glucocorticoid Receptor Signaling	3.05E-02	20

Nucleotide Excision repair	2.77E-04	8

#### i). Metabolic pathways

Out of 41 molecules involved in energy metabolism in cornea, 35 were upregulated by more than 2 fold indicating active metabolism in this region possibly related to cell division and turn over. Likewise, Karsten *et al *[69] have noted upregulated expression of oxidative phosphorylation, purine and protein metabolism in neural progenitor cells denoting increased energy consumption and high protein turn over due to active cellular processes like proliferation and cell migration. Cornea also had upregulated expression of related carbohydrate metabolic pathways such as pyruvate, citrate/glycolysis and gluconeogenesis. Cornea was also enriched for glutathione metabolism, which is crucial for maintaining corneal transparency, cell membrane integrity and protection against oxidative stress.

Studies on side population cells from various tissues have demonstrated that the energy consumption, transcription, translation and metabolism in the undifferentiated and quiescent cells are minimal [24,70]. Although oxidative phosphorylation, amino acid, carbohydrate and energy metabolism were found to be enriched in LEC, limbus and cornea, the gene expression in these pathways was downregulated in LEC and limbus, which supports the presence of undifferentiated and quiescent cells in these regions.

#### ii). Signalling pathways

All the three OS regions were enriched for NRF2 mediated oxidative stress response (table 4). It has a role in cell protection during cell cycling.

### SC signalling pathways in the OS

**Figure 4:**
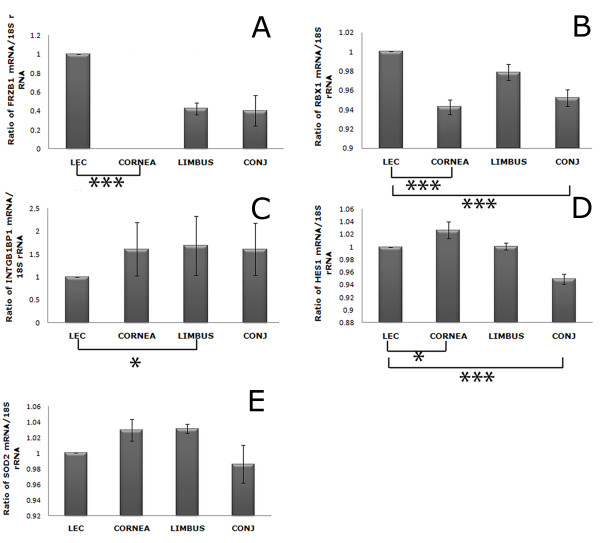
**Composite image of graphs of Real Time PCR performed on genes of interest**. The real time PCR was performed on OS epithelial regions of LEC, cornea, limbus and conjunctiva comprising of three biological replicates (3 eyes) with four sets of technical replicates A, B, C & D. These were processed in triplicate. The cycle threshold (Ct) value for these samples were averaged and normalised with 18S rRNA Ct values. Significant p values between LEC and other OS regions is shown as (*),*p < 0.05, ***p < 0.001. Data are expressed as means +/- standard errors of the mean (SEM).

Fevr *et al *have demonstrated the importance of WNT receptor-beta catenin signalling pathway in maintaining intestinal crypt structures and SC in their niche [71]. It is also crucial for maintaining HSC in a quiescent state and also has a role in SC self renewal [72]. We had found upregulated expression of molecules, involved in WNT receptor-beta catenin signalling such as LEF1, and CDH1 in LEC. Soluble WNT antagonists (sWA) maintain skin bulge SC quiescence[73] and also contribute to SC pool maintenance in gastric tissue[41]. FRZB1, a soluble WNT antagonist (sWA) was uniquely expressed in LEC. FRZB1 was weakly expressed in microarrays but real-time PCR and immunofluorescence results showed high gene and protein expression of FRZB1 in LEC compared to other regions (Figure 4A, 5F). Planar polar component of WNT pathway, involved in regulation of cell adhesion and motility, was upregulated in cornea only (table 5). Genes related to the Notch [74], Jak/STAT [75], TGF-Beta/BMP [76] and the Hedgehog (HH) signalling pathways involved in regulation of cell proliferation and differentiation in response to cytokines and growth factors; were found to be upregulated in cornea but not in LEC (table 5). HES1, a target gene of Notch signalling pathway, and RBX1 a TGF-Beta gene were further validated with real time PCR.

**Figure 5:**
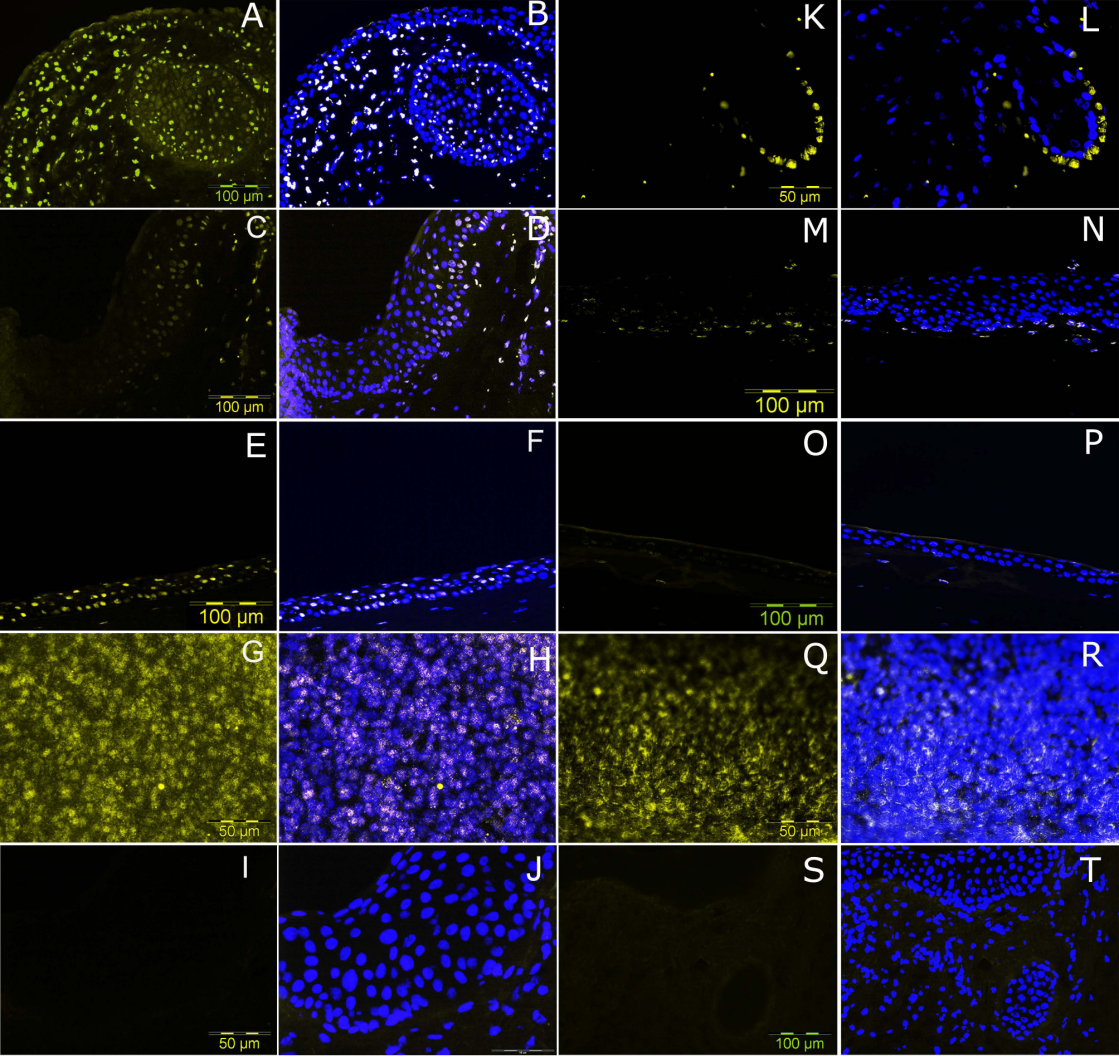
**Immuno-fluorescent staining for expression of stem cell molecules on ocular surface epithelium**. Immunofluorescence with HES1 and FRZB1 was performed on radial cut sections of LEC (A, B, K, L), limbus (C, D, M, N) cornea (E, F, O, P) and tonsil (G, H, Q, R). Images (K, L, G, H, Q, R, I, J) are at 40× magnification, the remainder are at 20× magnification (scale bars shown). HES1 immunofluorescence co-localised with DAPI nuclear dye is shown in images (B. D, F, H,). Images (A, C, E, G) show HES1 (TRITC) staining. FRZB1 immunofluorescence co-localised with DAPI dye is shown in images (L, N, P, R). Images (K, M, O, Q,) are of FRZB1 with TRITC dye. Positive staining for HES1 and FRZB1 (B, D, F, H), (N, P, R) is seen as whitish nuclear stain, due to co-localisation of FRZB1 and HES1 (TRITC dye) and nuclear stain DAPI in the cell nuclei. FRZB1 immunofluorescence of LEC (L) is seen as yellow colouration as it intensely stained the nuclei of the basal epithelial cells in the LEC and masked the underlying DAPI staining. Positive nuclear staining of Tonsil with HES1 antibody (G, H) has speckled appearance and FRZB1 (Q, R) appears as a rim around the cell nucleus. Images (I, J) and (S, T) are negative controls for HES1 and FRZB1 antibody respectively.

**Table 5:** Stem cell signaling pathway genes in OS epithelial regions

Accession numbers	Gene symbols	Fold change		
		
		LEC	Limbus	Cornea
**WNT signalling pathway**				

NM_016269	LEF1	(1.2)	-	-

NM_006261	CDH1	(5.3)	-	(-5.8)

NM_013263	BRD7	(-4.0)	(1.8)	(4.0)

NM_001892	CSNK1A1	-	(4.6)	-

AF267864	TBL1XR1	-	-	(1.9)

NM_003507	FZD7	-	-	(2.0)

NM_022825	PORCN	-	-	(2.0)

NM_018890	RAC1	(-3.0)	-	(3.0)

NM_001320	CSNK2B	(-2.6)	-	(2.6)

NM_014248	RBX1	(5.6)		(-5.6)

**Notch Signalling pathway**				

NM_014276	RBPJL	(-3.9)	-	(3.9)

NM_005524	HES1	(-1.5)	-	(2.1)

NM_203283	RBPJ	-	-	(4.6)

NM_033632	FBXW7	(-6.1)	-	(6.1)

**JAK/STAT Signalling pathway**				

NM_004232	SOCS6	-	-	(3.9)

U52912	LEPR	(-3.4)	(-2.2)	(3.4)

U52914	LEPR	-	-	(2.3)

AJ271736	IL9R	-	(-4.2)	(4.2)

NM_000565	IL6R	-	(-8.7)	(8.7)

NM_003150	STAT3	-	-	(1.7)

NM_000297	PKD2	(-2.8)	-	(2.8)

NM_014432	IL20RA	(-4.2)	-	(4.2)

**TGF-Beta Signalling pathways**				

NM_002165	ID1	(-3.6)	(1.5)	(3.6)

NM_006350	FST	-	-	(2.4)

NM_014248	RBX1	(5.6)	-	(-5.6)

NM_002165	ID1	(-3.6)	(1.5)	(3.6)

**BMP Signalling pathway**				

AL096865	RUNX2	(-2.4)	-	(2.4)

NM_006350	FST	-	-	(2.4)

**Hedgehog signalling pathway (HH)**				

NM_001892	CSNK1A1	-	(4.5)	-

NM_016004	IFT52	-	(2.5)	-

NM_005870	SAP18	(-5.3)	(-3.7)	(6.2)

The differentially expressed SC signalling molecules in the OS epithelial regions were identified with KEGGS pathways and reactome. Numbers in brackets are fold change. Negative values indicate downregulated and positive values are upregulated gene expression. No values denote absence of gene expression in the region.

### Comparison of gene expression in OS epithelium with other SC populations

LEF1 was found to be crucial for maintaining stemness across various SC populations such as embryonic [77], mesenchymal [44] and epithelial [36] SC. In this study it was uniquely upregulated in LEC. Myc genes encode for transcription factors, which activate genes influencing cell proliferation, cell growth, apoptosis and SC self-renewal. A study on epidermal SC has noted upregulated expression of myc genes in α6+/MHCI^+ ^cells. These cells have characteristic features of TAC. However, myc genes were found to be downregulated in α6+/MHCI^- ^population of cells consisting of quiescent SC [78]. Myc genes such as *ANXA1, TGFB1, FTH1, VAMP8, KRT5, HSPB1 and UGCG *were upregulated in cornea and TXN (4.5) in limbus but these genes were downregulated in LEC. Self renewal genes such as *FZD7 *[44,78-80], *PCNA *[69], and *STAT3 *[77,81-83] expressed in PSC populations (haematopoietic, mesenchymal, epithelial, neuronal and embryonic SC) were also upregulated in cornea.

### Comparison with other ocular gene expression studies

We had noted some similarities with other OS gene expression studies, particularly with regards to KRT19, an epithelial SC marker previously identified in limbus [11]. KRT19 was found to be upregulated in LEC and limbal epithelium in this study. KRT13 a suprabasal epithelial marker [23], was also upregulated in limbus. A study comparing the gene profile of limbal and corneal basal cells in mice has noted preferential expression of epithelial SC genes such as FGFR1 (Fibroblast Growth Factor Receptor 1) and S100A6 (S100 calcium binding protein A6) in limbus [23], however we had noted upregulation of these molecules in the corneal epithelium. This difference could be related to an interspecies variation. We had also noted upregulated expression of *CRTAC1, CTSL2, NQO1, KRT12, MAL, IGFBP6, IGFBP7, S100A10 *in the cornea. These genes were previously identified by Turner et al in their oligonucleotide microarray study on corneal and conjunctival epithelium [84]

### Validation of microarray data

#### i) Quantitative real-time PCR (qPCR)

Relative quantification of genes of interest (*FRZB1, RBX1, INTGB1BP1, HES1, SOD2*) was performed with real time PCR on OS regions such as limbus, cornea, and conjunctiva in comparison with LEC (figure 4). FRZB1 was found to be significantly expressed in LEC but was absent in all corneal replicates and insignificantly expressed in limbus and conjunctiva (figure 4A). RBX1 was significantly expressed in LEC compared to cornea and conjunctiva (figure 4B). Significant expression of ITGB1BP1 was noted between limbus and LEC with least expression in LEC (figure 4C). Significant expression of HES1 was noted between LEC and cornea and also between LEC and conjunctiva (Figure 4D). Although SOD2 was significantly expressed in all the OS regions posthoc analysis had failed to demonstrate any significant relationship between the groups (Figure 4E).

#### ii) Immuno fluorescence

Immunofluorescence of frozen tissue sections of OS epithelium was performed with *FRZB1 *and *HES1 *antibodies (Figure 5). A previous study on HES1 expression in mice corneal epithelial stem/progenitor cells has demonstrated that an increased expression of HES1 in these cells was crucial for regulation of corneal development and homeostasis [85]. Intense nuclear staining of *HES1 *was noted in LEC and the stromal cells adjacent to LEC (figure 5A) as compared to limbus (figure 5B). This evidence is supportive of increased proportion of stem progenitor cells in LEC and also in the surrounding LEC stroma. Few cells in the cornea along the basal epithelium also expressed *HES1 *(Figure 5C). Nuclear staining with FRZB1 was prominently seen in the basal epithelium of the LEC (Figure 5F) and in some areas of the limbus (Figure 5G). Figure 5H shows absence of *FRZB1 *expression in corneal epithelium.

In summary, gene ontology and gene expression patterns noted in this study are suggestive of LEC being the most metabolically dormant and undifferentiated region as compared to cornea and limbus. LEC was enriched for GO terms related to quiescent adult SC. Upregulated genes in LEC such as *CDH1, SERPINF1, LEF1, FRZB1, KRT19, SOD2, EGR1 *along with Beta catenin-WNT signalling pathway are known to be involved in SC maintenance. However limbus and cornea had presence of a mixed population of stem/progenitor, and differentiated cells. Similar to LEC limbus was metabolically dormant, but also had upregulated SC signalling molecules related to WNT, TGF-Beta and Hedgehog pathways. Cornea had upregulated gene expression related to cell cycling, self renewal, proliferation, cell differentiation growth factors, cytokines and SC signalling genes in the WNT, Notch, TGF-Beta pathways. This further strengthens the evidence for the presence of long term surviving early TACs (transient cells) with self renewal capacity in the cornea.

## Conclusion

This study is the first to characterise the *in situ *gene expression profile of laser microdissected LEC and demonstrate the presence of two distinct SC compartments on the OS. We have demonstrated, at the transcriptome level that the LEC has features that appear to be consistent with that of a quiescent SC niche. Although the limbus was metabolically dormant it had a mixed population of differentiated and undifferentiated cells. Our study clearly demonstrates that cornea is the most differentiated and proliferating region. The gene expression of this region is also suggestive of the presence of early TACs with self renewal capacity (transient cells) Clinical evidence supports our findings that cornea has the potential to sustain steady state turnover of its epithelium in healthy ocular conditions and LEC is the potential reservoir of limbal SC. Although a specific limbal SC marker has yet to be elucidated, our findings have identified several genes of interest, which will be further studied as candidate genes to validate their potential as stem cell markers.

## Methods

### Donor eye tissue preparation

This study was carried out at the Queens Medical Centre, University Hospital Nottingham, England with approval of Nottingham Research Ethics Committee (REC NO: OY030202). Protocol was consistent with Tenets of Declaration of Helsinski. Informed, written consent was obtained from relatives of all the donors. The eyes were harvested within 48 hours of death under aseptic conditions using conventional techniques in order to maintain the RNA viability. Four pairs of human donor eyes were collected for microarray study. The inclusion criteria were: i) donors aged between 20 to 70 years; ii) donors of either sex; iv) Eyes with intact and undamaged OS epithelium, confirmed with dissecting microscope and patient history as ascertained from the case notes. The corneoscleral button was dissected from the cadaver eye and processed for sectioning using established techniques within our department [12]. Briefly, a 15 mm corneal button was trephined maintaining a 3 mm frill of conjunctiva around the limbus and divided into eight triangular radial segments. Each segment was positioned in the optimum temperature compound (OCT, Emitech Ltd, East Sussex, England) with the long dissected edge parallel to the OCT surface and then gradually frozen using isopentane precooled in liquid Nitrogen. The frozen tissue blocks were stored at -80°c for future cryosectioning.

### Processing of Standard probe samples

Depending on the number of biological samples and their replicates sufficient amount of control samples are required for any microarray study. As it was not possible to generate required amount of control sample with LMD, we followed the reference probe hybrid approach for sample processing as described by Neal and Westwood [86]. Briefly, the reference samples were prepared by pooling the corneal and conjunctival epithelial RNA. The tissue for RNA extraction was obtained by scrapping the OS epithelial regions from the cadaver eyes. This approach generated sufficient amount of reference samples for the microarrays, facilitated better comparison between the two regional arrays and also highlighted the variations in gene expression between the biological replicates but not the reference samples. For real time PCR, RNA extraction for each OS region was performed in triplicate from three different cadaver eyes using RNeasy Microkit (Qiagen, Crawley, West Sussex, UK) according to manufacturer's protocol.

### Laser Microdissection (LMD)

Under RNase and DNase free conditions, 6-7 μm serial sections of frozen tissue blocks of corneoscleral buttons were prepared with Jung CM 1900 cryostat (Leica, UK) and examined under light microscope for presence of LEC and also for good epithelial histology of other OS regions. Prior to LMD, sections were placed on poly-L-lysine coated PALM^® ^membrane slides, fixed in precooled 70% v/v ethanol for 5 minutes and air dried. Following which, the sections were briefly stained with 0.1% w/v Toludine Blue for 30 seconds, washed in DEPC treated water and air dried. LMD was performed with the PALM^® ^Microbeam systems (Zeiss Instruments, Bernreid, Germany), using the Robo LPC laser function, according to manufacturer's recommended guidelines. The area of interest was cut and catapulted in the caps of collection tubes, coated with special adhesive. Thereafter RLT RNA lysis buffer (QIAGEN) was added to the collection tubes and stored at -80°c until further use. For each of the 4 eyes, multiple LMD samples were collected from five regions creating 5 independent experimental groups; 1) LEC; 2) limbus; 3) cornea; 4) LEC stroma, and 5) conjunctiva.

### Total RNA Extraction

For microarrays total RNA extraction from LMD sections for samples and reference sample was performed with RNeasy kit, including DNase treatment, according to manufacturer's protocols (QIAGEN House, West Sussex, UK). RNA quantity and quality was measured with Picoassay 2100 Bioanalyzer (Agilent Technologies, USA). Samples with concentration ranging between 20-90 pg/μl and an average RIN value (RNA Integrity Number) of 5.1 [32] were used for further analysis.

### Preparation of reference sample for Standard Probe

1 μg of total RNA from each corneal and conjunctival reference sample was mixed and ethanol precipitated by adding 0.1 volumes Sodium Acetate and 2.5 volumes 100% ETOH followed by incubation at -20°c for 30 minutes. Samples were then centrifuged at 21000 × g at 4°c for 15 minutes and the pellet washed in 250 μl of 70% v/v ETOH before recentrifugation at 21000 × g for 5 min. The RNA pellet was dried and resuspended in 10 μl ultra pure, RNase free water followed by quantitation using NanoDrop ND-1000 UV-Vis Spectrophotometer (Labtech International Ltd-UK).

### RNA Amplification, labelling of the samples and reference sample

Each RNA sample and reference sample was further processed for microarray analysis. To 0.2 ng/10 μl of starting RNA, 0.5 μl of 1:5000 diluted spike control (GE Healthcare life Sciences Universal Score card oligonucleotide control), which are sequences from *E. coli *genes, was added for validation of microarray data. Complimentary RNA (cRNA) amplification was performed with Amino Allyl Message Amp™ II aRNA Amplification kit and labelled with Cy3 and Cy5 reactive dye [Ambion, (Europe) LTD, UK], the Frequency of Incorporation (FOI) of the Cy3 and Cy5 dye in the labelled samples was measured, according to manufacturer's protocols. Quality control of the purified cRNA samples was performed at the end of 1^st ^and 2^nd ^rounds by NanoDrop spectrophotometer. Unsatisfactorily amplified and labelled samples were discarded and new samples were processed.

### Microarray hybridisation

500 ng of a labelled sample and reference sample with matching FOI were separately blocked with 2 μl Poly A and 2 μl human Cot 1 DNA and then combined. This was followed by ethanol precipitation to generate 2 μl of the hybrid probe in nuclease free water. Prior to hybridisation, the 30K Human spotted oligonucleotide glass arrays manufactured in house, (Post-Genomics technologies facility, University of Nottingham, UK), were blocked with appropriate buffers (5× SSC, 0.2% w/v SDS, 1% w/v BSA), (SSC buffer: Saline, Sodium Citrate buffer; SDS buffer: Sodium Dodecyl. Sulphate; BSA: Bovine Serum Albumin). The slides were then washed thrice with ultra pure water, 100% ETOH and spun dried. For hybridisation, 100 μl of pre warmed Schott 1× Hybridisation Buffer was gently added to the hybrid probe, heated to 95°C, 2 minutes followed by hybridisation on automated hybridisation station TECAN HS 4800 (Tecan UK Ltd) using manufacturers protocols. The conditioned hybridisation station was primed with ultra pure water followed by a 1 min and a 15 min wash in 5× SSC, 0.2% w/v SDS, at 50°C. Next, 100 μl of probe was injected onto the slide and hybridised with agitation at 50°C for 16 hours, followed by sequential washing in four cycles of 2× SSC, 0.1% SDS, 2 cycles of 1× SSC, 0.1% SDS, and 0.1× SSC, 0.2% SDS at 40°C, 2 cycles of 0.1× SSC at 23°C, followed by a final cleaning cycle of ultrapure water before drying. On completion of the programme, the slides were covered to protect against light and scanned immediately.

### Scanning and Data analysis

Hybridised microarray slides were scanned to obtain two coloured digital images on an Agilent BA scanner. The images were further analysed with Gene Pix Pro 6.0 software. Poor spots and spots overlying areas of high background intensities were lassoed and removed from further analysis. However, spots of varying sizes but with good intensities were included in analysis. The raw data was uploaded on *BASE *(Bio Array Software Environment; (Lund University, Lund, Sweden) which is a MIAME (Minimum Information about a Microarray Experiment) compliant system [87]. The raw data sets were expressed as log ratio of channel 1/channel 2 intensities or log ratio of Cy5/Cy3 (sample/standard probe). Bioassay sets created from the raw data sets were further filtered to refine the data by removing 'noise'. Following filtrations, intra-array Lowess normalisation was performed on *BASE*, and the bioassay sets were then imported to *J-Express Pro *software (http://www.molmine.com; MolMine AS, Norway). In *J-Express *inter array scale normalisation was performed on the imported data sets. Further statistical analysis of the data was performed in J-Expresspro using a feature subset selection algorithm (FSS) for two unpaired groups comparison with following parameters: P value was selected for score method, individual ranking of the genes was performed and fold change values were log(2) transformed.

### Significance Analysis of Microarrays (SAM)

Differentially expressed gene list was generated from the FSS derived gene list (p value ≤ 0.05) by performing SAM on the data to determine the fold change and the False Discovery Rate (FDR). Cutoff limit of FDR was set to ≤ 5% and genes with FDR above this limit were excluded from the analysis. Following SAM the five individual gene lists generated for each of the OS regions were merged to form an overlapping gene list for each region.

### Principal Component Analysis (PCA)

PCA evaluates variation between the samples. The 2D plots representing the samples (Figure 2 left) and the genes (Figure 2 right) were generated following FSS analysis in J-Expresspro. The variance of axes was displayed in percentage. The data with greatest variation is clustered at the first principal component axis.

### Gene Ontology (GO)

The enriched GO terms for each region was determined by uploading the significant gene list for each region individually on the Database for Annotation, Visualization, and Integrated Discovery (*DAVID*) v6.7, 2008, (http://david.abcc.ncifcrf.gov) and the data was analysed according to published methodology [88,89]. GO terms associated with the biological processes (GO~BP) were mostly considered for analysis. Statistical significance of the GO terms was established by Fishers Exact T test. GO terms with p values < 0.05 were considered statistically significant.

### Canonical pathways on Ingenuity Pathway Analysis

The microarray data was also analysed with Ingenuity Pathways Analysis (IPA) *version 7.6 *(Ingenuity^® ^Systems, http://www.ingenuity.com). IPA identified the canonical signalling and metabolic pathways from the IPA library that were most significant to the data set. The significance of the association between the data set and the canonical pathway was measured as a ratio and the p value calculated with Fischer Exact test. The data discussed in this publication have been deposited in NCBI's Gene Expression Omnibus and are accessible through GEO Series accession number GSE19035 http://www.ncbi.nlm.nih.gov/geo/query/acc.cgi?acc=GSE19035.

### Quantitative Gene Expression Analysis (Real-time PCR)

The relative quantification of mRNA for genes of interest on OS epithelium (LEC, limbus, cornea, and conjunctiva) was performed with real-time PCR. Approximately 1 ng/μl concentration of RNA was used for cDNA synthesis using QuantiTect Reverse Transcription Kit (QIAGEN) according to manufacturer's protocol. Inventoried Taqman assays (Applied Biosystems, Foster City, CA) were used for selected genes of interest. Each reaction was performed in triplicate with final reaction volume of 20 μl. The reaction components comprised of, 10 μl 2× Taqman Gene Expression Master Mix (Applied Biosystems), 1 μl of 20× Taqman Assay probes (Applied Biosystems), 1 μl cDNA (1:2 dilutions), 8 μl nuclease free water (Promega UK, Southampton, UK). Non template, reverse transcriptase negative and positive cDNA from Universal Human Reference RNA (Stratagene, La Jolla, CA) were run as controls. Amplification was performed on the Mx3005P multicolour 96 well PCR system (Stratagene Europe, Amsterdam, Netherlands) with the following parameters, 50°C for 2 min and then 95°C for 10 min followed by 45 cycles of 95°C for 15 seconds and 60°C for 1 minute. Data analysis was performed on Mxpro ver 4.2 software to measure the threshold cycle (Ct) for each reaction. Set of triplicate Ct values for each sample was averaged. Normalisation of the average sample gene expression was performed with the average Ct value of 18S rRNA endogenous gene control for that sample.

### Statistical Analysis of Real time PCR Data

The real time PCR data was statistically analysed on SPSS *ver *16. The average normalised Ct values of gene of interest for each of the OS regions were subjected to Levene's test, to measure the equality of variance. The normally distributed data was then analysed with one way analysis of variance (ANOVA) with Bonferonni's posthoc test. Non parametric distributed samples were analysed by Kruskal-Wallis test followed by Mann-Whitney test, p value < 0.05 was considered statistically significant.

### Immunohistochemistry

Six micron frozen tissue sections mounted on 2% 3-aminopropyltriethoxysilane (APES), (SIGMA) coated slides were fixed in acetone followed by three washes with wash solution consisting of 1%PBS, 1% BSA and 0.1% v/v Triton-X for 5 minutes each. The sections were then blocked using 10% v/v goat serum (Invitrogen, UK), followed by over night incubation with primary antibody prepared in the above mentioned wash solution at 4°C. Primary antibodies were rabbit anti human polyclonal FRZB (H-170), dilution, 1:100 (Santa Cruz Biotechnology, INC Europe); and HES1, rabbit anti human, dilution 1:100, (US Biological, USA). Slides were washed 3 times for 5 minutes in wash solution followed by detection of the primary with secondary antibody Goat anti-rabbit conjugated Alexa Fluor 555 (Dilution 1:300; Invitrogen Ltd, Paisley, Scotland) for 30 minutes. This was followed by washes with above mentioned wash solution for 5 minutes and then counterstained with nuclear stain DAPI (4', 6-diamidino-2-phenylindole) (Dilution: 25 μl in 5 ml PBS of stock solution 1 mg in 4 ml PBS, Santacruz Biotechnology, UK) for 4 minutes followed by further washing. Slides mounted with fluorescent mounting medium (Dako, Ely, UK) were visualised using the B51X fluorescent microscope with software CELL^^^F (Olympus UK Ltd, UK).

## Authors' contributions

BBK was involved in sample collection and processing and also performed microarray, real-time PCR and immunohistochemistry experiments and its analysis. HSD and PJT helped and supervised over the analysis and upload of microarray data on GEO profile. HSD conceived the study and HSD, PJT, BBK, DGP, AH and VAS participated in its design. HSD and AH had supervisory role and looked after the administration and financial aspect of the project. BBK created a draft of the manuscript, HSD, PJT, AH, and IM helped in editing the manuscript. DGP is supervisor for lasermicrodissection and advised on performing the procedure. IM had advised on data analysis and trouble shooting of real time PCR experiments. AMY helped with immunofluorescence experiment. All authors have read and approved the manuscript.
